# (*E*)-*N*′-[1-(4-Bromo­phen­yl)ethyl­idene]-2-hydroxy­benzohydrazide

**DOI:** 10.1107/S1600536809042081

**Published:** 2009-10-17

**Authors:** Chuan-Gang Fan, Ming-Zhi Song

**Affiliations:** a College of Chemistry and Chemical Technology, Binzhou University, Binzhou 256600, Shandong, People’s Republic of China

## Abstract

In the title compound, C_15_H_13_BrN_2_O_2_, the two aromatic rings form a dihedral angle of 7.9 (1)° and an intra­molecular N—H⋯O hydrogen bond influences the mol­ecular conformation. In the crystal, inter­molecular O—H⋯O hydrogen bonds link the mol­ecules into chains propagated in [001]. The crystal packing exhibits also π–π inter­actions, which pair mol­ecules into centrosymmetric dimers with short inter­molecular distances of 3.671 (4) Å between the centroids of aromatic rings.

## Related literature

For the biological properties of Schiff base ligands, see: Jeewoth *et al.* (1999 ▶). For related structures, see: Fun *et al.* (2008 ▶); Cui *et al.* (2009 ▶); Nie (2008 ▶).
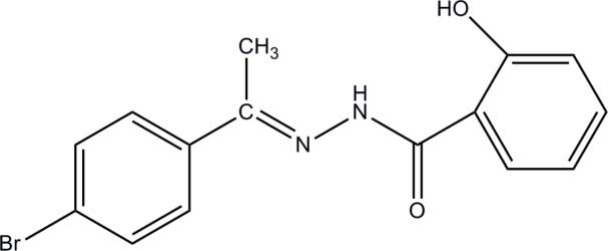


## Experimental

### 

#### Crystal data

C_15_H_13_BrN_2_O_2_
*M* *_r_* = 333.18
Monoclinic, 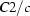

*a* = 27.805 (3) Å
*b* = 7.9061 (9) Å
*c* = 13.5002 (15) Å
β = 113.344 (2)°
*V* = 2724.8 (5) Å^3^
*Z* = 8
Mo *K*α radiation
μ = 3.02 mm^−1^
*T* = 298 K
0.39 × 0.14 × 0.12 mm

#### Data collection

Siemens SMART APEX CCD area-detector diffractometer
Absorption correction: multi-scan (*SADABS*; Sheldrick, 1996 ▶) *T*_min_ = 0.386, *T*_max_ = 0.713
6480 measured reflections
2397 independent reflections
1602 reflections with *I* > 2σ(*I*)
*R*_int_ = 0.071

#### Refinement

*R*[*F* ^2^ > 2σ(*F* ^2^)] = 0.045
*wR*(*F* ^2^) = 0.111
*S* = 0.95
2397 reflections
181 parameters
H-atom parameters constrained
Δρ_max_ = 0.54 e Å^−3^
Δρ_min_ = −0.74 e Å^−3^

### 

Data collection: *SMART* (Siemens, 1996 ▶); cell refinement: *SAINT* (Siemens, 1996 ▶); data reduction: *SAINT*; program(s) used to solve structure: *SHELXS97* (Sheldrick, 2008 ▶); program(s) used to refine structure: *SHELXL97* (Sheldrick, 2008 ▶); molecular graphics: *SHELXTL* (Sheldrick, 2008 ▶); software used to prepare material for publication: *SHELXTL*.

## Supplementary Material

Crystal structure: contains datablocks I, global. DOI: 10.1107/S1600536809042081/cv2623sup1.cif

Structure factors: contains datablocks I. DOI: 10.1107/S1600536809042081/cv2623Isup2.hkl

Additional supplementary materials:  crystallographic information; 3D view; checkCIF report

## Figures and Tables

**Table 1:** Hydrogen-bond geometry (Å, °)

*D*—H⋯*A*	*D*—H	H⋯*A*	*D*⋯*A*	*D*—H⋯*A*
N1—H1⋯O2	0.86	1.95	2.637 (3)	136
O2—H2⋯O1^i^	0.82	1.86	2.677 (3)	178

Symmetry code: (i) 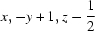.

**Fig. 1.**
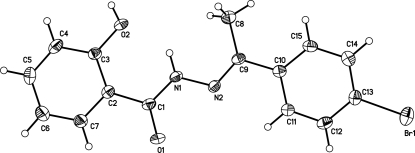
The molecular structure of (I) showing the atomic numbering scheme and 30% probability displacement ellipsoids.

## supplementary crystallographic information

### Comment

Schiff base compounds have received considerable attention during the last decades due to their structures and biological properties (Jeewoth *et al.*, 1999). We report here the crystal structure of the title Schiff base compound (I).

In (I) (Fig. 1), the bond lengths and angles are normal and comparable to the values observed in similar compounds (Nie, 2008; Fun *et al.*, 2008; Cui *et al.*, 2009). The C9=N2 bond length in the molecule is 1.282 (4) °, showing the double-bond character. The dihedral angle between the benzene ring C2-C7 and the benzene ring C10-C15 is 7.9 (1)°, indicating that  two these rings are approximately coplanar.

In the crystal, intermolecular O—H···O hydrogen bonds (Table 1) link the molecules into chains propagated in direction [001]. The crystal packing exhibits also π···π interactions, which pair molecules into centrosymmetric dimers with short intermolecular distance of 3.671 (4) Å between the centroids of aromatic rings.

### Experimental

Salicyloyl hydrazide (5.0 mmol), 20 ml e thanol and 4-bromoacetophenone (5.0 mmol) were mixed in 50 ml flash. After refluxing 3 h, the resulting mixture was cooled to room temperature, and recrystallized from ethanol. Elemental analysis: calculated for C_15_H_13_BrN_2_O_2_: C 54.07, H 3.93, N 8.41%; found: C 54.21, H 3.85, N 8.52%.

### Refinement

All H atoms were placed in geometrically idealized positions (O—H 0.82, N—H 0.86 and C—H = 0.93–0.96 Å) and treated as riding, with *U*_iso_(H) = 1.2U-1.5U_eq_ of the parent atom.

### Crystal data

**Table:**

C_15_H_13_BrN_2_O_2_	*F*(000) = 1344
*M**_r_* = 333.18	*D*_x_ = 1.624 Mg m^−^^3^
Monoclinic, *C*2/*c*	Mo *K*α radiation, λ = 0.71073 Å
*a* = 27.805 (3) Å	Cell parameters from 1955 reflections
*b* = 7.9061 (9) Å	θ = 2.7–26.0°
*c* = 13.5002 (15) Å	µ = 3.02 mm^−^^1^
β = 113.344 (2)°	*T* = 298 K
*V* = 2724.8 (5) Å^3^	Block, colourless
*Z* = 8	0.39 × 0.14 × 0.12 mm

### Data collection

**Table:**

Siemens SMART APEX CCD area-detector diffractometer	2397 independent reflections
Radiation source: fine-focus sealed tube	1602 reflections with *I* > 2σ(*I*)
graphite	*R*_int_ = 0.071
phi and ω scans	θ_max_ = 25.0°, θ_min_ = 1.6°
Absorption correction: multi-scan (*SADABS*; Sheldrick, 1996)	*h* = −32→32
*T*_min_ = 0.386, *T*_max_ = 0.713	*k* = −5→9
6480 measured reflections	*l* = −16→14

### Refinement

**Table:**

Refinement on *F*^2^	Primary atom site location: structure-invariant direct methods
Least-squares matrix: full	Secondary atom site location: difference Fourier map
*R*[*F*^2^ > 2σ(*F*^2^)] = 0.045	Hydrogen site location: inferred from neighbouring sites
*wR*(*F*^2^) = 0.111	H-atom parameters constrained
*S* = 0.95	*w* = 1/[σ^2^(*F*_o_^2^) + (0.0507*P*)^2^] where *P* = (*F*_o_^2^ + 2*F*_c_^2^)/3
2397 reflections	(Δ/σ)_max_ = 0.002
181 parameters	Δρ_max_ = 0.54 e Å^−^^3^
0 restraints	Δρ_min_ = −0.74 e Å^−^^3^

### Special details

**Table:**

Geometry. All e.s.d.'s (except the e.s.d. in the dihedral angle between two l.s. planes) are estimated using the full covariance matrix. The cell e.s.d.'s are taken into account individually in the estimation of e.s.d.'s in distances, angles and torsion angles; correlations between e.s.d.'s in cell parameters are only used when they are defined by crystal symmetry. An approximate (isotropic) treatment of cell e.s.d.'s is used for estimating e.s.d.'s involving l.s. planes.
Refinement. Refinement of *F*^2^ against ALL reflections. The weighted *R*-factor *wR* and goodness of fit *S* are based on *F*^2^, conventional *R*-factors *R* are based on *F*, with *F* set to zero for negative *F*^2^. The threshold expression of *F*^2^ > σ(*F*^2^) is used only for calculating *R*-factors(gt) *etc*. and is not relevant to the choice of reflections for refinement. *R*-factors based on *F*^2^ are statistically about twice as large as those based on *F*, and *R*- factors based on ALL data will be even larger.

### Fractional atomic coordinates and isotropic or equivalent isotropic displacement parameters (Å^2^)

**Table:**

	*x*	*y*	*z*	*U*_iso_*/*U*_eq_	
Br1	1.171700 (16)	1.05278 (6)	1.41157 (3)	0.0585 (2)	
N1	0.92749 (11)	0.6137 (4)	0.9225 (2)	0.0342 (8)	
H1	0.9291	0.6170	0.8602	0.041*	
N2	0.96688 (11)	0.6843 (4)	1.0103 (2)	0.0325 (7)	
O1	0.88354 (11)	0.5298 (4)	1.02232 (19)	0.0555 (9)	
O2	0.88245 (10)	0.5567 (4)	0.71317 (18)	0.0434 (7)	
H2	0.8821	0.5286	0.6545	0.065*	
C1	0.88626 (13)	0.5393 (5)	0.9337 (3)	0.0329 (9)	
C2	0.84455 (14)	0.4681 (5)	0.8353 (3)	0.0317 (9)	
C3	0.84277 (13)	0.4765 (5)	0.7298 (3)	0.0325 (9)	
C4	0.80124 (15)	0.4043 (6)	0.6450 (3)	0.0450 (11)	
H4	0.7998	0.4127	0.5752	0.054*	
C5	0.76224 (15)	0.3206 (6)	0.6629 (3)	0.0467 (11)	
H5	0.7347	0.2722	0.6053	0.056*	
C6	0.76374 (15)	0.3079 (5)	0.7662 (3)	0.0461 (11)	
H6	0.7376	0.2496	0.7787	0.055*	
C7	0.80399 (14)	0.3818 (5)	0.8496 (3)	0.0394 (10)	
H7	0.8044	0.3744	0.9187	0.047*	
C8	1.01117 (15)	0.7562 (6)	0.8889 (3)	0.0453 (11)	
H8A	1.0056	0.6454	0.8573	0.068*	
H8B	1.0457	0.7943	0.9003	0.068*	
H8C	0.9858	0.8331	0.8413	0.068*	
C9	1.00561 (13)	0.7498 (5)	0.9952 (2)	0.0314 (9)	
C10	1.04721 (13)	0.8237 (5)	1.0939 (3)	0.0312 (9)	
C11	1.04697 (14)	0.7866 (5)	1.1949 (3)	0.0387 (10)	
H11	1.0216	0.7137	1.1993	0.046*	
C12	1.08322 (15)	0.8551 (6)	1.2876 (3)	0.0441 (10)	
H12	1.0822	0.8297	1.3540	0.053*	
C13	1.12116 (14)	0.9617 (5)	1.2819 (3)	0.0376 (9)	
C14	1.12300 (16)	0.9996 (6)	1.1834 (3)	0.0446 (10)	
H14	1.1487	1.0713	1.1795	0.054*	
C15	1.08594 (14)	0.9290 (5)	1.0911 (3)	0.0393 (10)	
H15	1.0873	0.9537	1.0248	0.047*	

### Atomic displacement parameters (Å^2^)

**Table:**

	*U*^11^	*U*^22^	*U*^33^	*U*^12^	*U*^13^	*U*^23^
Br1	0.0516 (3)	0.0628 (4)	0.0480 (3)	−0.0060 (3)	0.0059 (2)	−0.0110 (2)
N1	0.0355 (17)	0.045 (2)	0.0228 (15)	0.0001 (16)	0.0125 (13)	−0.0005 (14)
N2	0.0320 (17)	0.039 (2)	0.0259 (16)	0.0008 (15)	0.0105 (14)	−0.0028 (14)
O1	0.0562 (18)	0.088 (3)	0.0281 (14)	−0.0174 (17)	0.0231 (13)	−0.0038 (15)
O2	0.0502 (16)	0.060 (2)	0.0236 (12)	−0.0083 (15)	0.0181 (12)	−0.0062 (12)
C1	0.036 (2)	0.038 (2)	0.0268 (19)	0.0080 (19)	0.0146 (16)	0.0022 (17)
C2	0.0311 (19)	0.032 (2)	0.0301 (19)	0.0081 (18)	0.0104 (16)	0.0016 (17)
C3	0.034 (2)	0.035 (2)	0.0305 (19)	0.0059 (18)	0.0151 (17)	0.0000 (17)
C4	0.050 (3)	0.054 (3)	0.027 (2)	0.003 (2)	0.0108 (19)	−0.0065 (19)
C5	0.034 (2)	0.048 (3)	0.048 (2)	−0.001 (2)	0.0052 (19)	−0.009 (2)
C6	0.038 (2)	0.049 (3)	0.053 (3)	−0.002 (2)	0.020 (2)	−0.002 (2)
C7	0.037 (2)	0.044 (3)	0.036 (2)	0.001 (2)	0.0145 (18)	0.0041 (19)
C8	0.048 (2)	0.054 (3)	0.039 (2)	−0.003 (2)	0.0221 (19)	−0.005 (2)
C9	0.037 (2)	0.032 (2)	0.0277 (19)	0.0079 (18)	0.0145 (17)	0.0014 (16)
C10	0.031 (2)	0.031 (2)	0.0338 (19)	0.0078 (18)	0.0158 (16)	0.0012 (17)
C11	0.040 (2)	0.044 (3)	0.035 (2)	−0.005 (2)	0.0175 (18)	−0.0008 (19)
C12	0.051 (2)	0.052 (3)	0.030 (2)	0.004 (2)	0.0164 (19)	0.0058 (19)
C13	0.032 (2)	0.036 (2)	0.041 (2)	0.0022 (19)	0.0106 (17)	−0.0046 (18)
C14	0.042 (2)	0.043 (3)	0.049 (2)	−0.005 (2)	0.018 (2)	−0.001 (2)
C15	0.043 (2)	0.044 (3)	0.036 (2)	0.000 (2)	0.0210 (18)	0.0068 (19)

### Geometric parameters (Å, °)

**Table:**

Br1—C13	1.899 (4)	C6—H6	0.9300
N1—C1	1.349 (5)	C7—H7	0.9300
N1—N2	1.374 (4)	C8—C9	1.504 (4)
N1—H1	0.8600	C8—H8A	0.9600
N2—C9	1.282 (4)	C8—H8B	0.9600
O1—C1	1.231 (4)	C8—H8C	0.9600
O2—C3	1.366 (4)	C9—C10	1.494 (5)
O2—H2	0.8200	C10—C15	1.374 (5)
C1—C2	1.485 (5)	C10—C11	1.398 (5)
C2—C7	1.395 (5)	C11—C12	1.369 (5)
C2—C3	1.407 (5)	C11—H11	0.9300
C3—C4	1.386 (5)	C12—C13	1.376 (5)
C4—C5	1.371 (5)	C12—H12	0.9300
C4—H4	0.9300	C13—C14	1.384 (5)
C5—C6	1.383 (5)	C14—C15	1.382 (5)
C5—H5	0.9300	C14—H14	0.9300
C6—C7	1.365 (5)	C15—H15	0.9300
			
C1—N1—N2	120.2 (3)	C9—C8—H8B	109.5
C1—N1—H1	119.9	H8A—C8—H8B	109.5
N2—N1—H1	119.9	C9—C8—H8C	109.5
C9—N2—N1	117.3 (3)	H8A—C8—H8C	109.5
C3—O2—H2	109.5	H8B—C8—H8C	109.5
O1—C1—N1	121.2 (3)	N2—C9—C10	114.8 (3)
O1—C1—C2	121.3 (3)	N2—C9—C8	125.2 (3)
N1—C1—C2	117.6 (3)	C10—C9—C8	120.1 (3)
C7—C2—C3	117.2 (3)	C15—C10—C11	117.5 (3)
C7—C2—C1	116.7 (3)	C15—C10—C9	123.4 (3)
C3—C2—C1	126.1 (3)	C11—C10—C9	119.1 (3)
O2—C3—C4	121.2 (3)	C12—C11—C10	121.5 (4)
O2—C3—C2	118.8 (3)	C12—C11—H11	119.3
C4—C3—C2	120.0 (3)	C10—C11—H11	119.3
C5—C4—C3	120.8 (3)	C11—C12—C13	119.7 (3)
C5—C4—H4	119.6	C11—C12—H12	120.2
C3—C4—H4	119.6	C13—C12—H12	120.2
C4—C5—C6	120.2 (4)	C12—C13—C14	120.5 (3)
C4—C5—H5	119.9	C12—C13—Br1	119.0 (3)
C6—C5—H5	119.9	C14—C13—Br1	120.5 (3)
C7—C6—C5	119.2 (4)	C15—C14—C13	118.8 (4)
C7—C6—H6	120.4	C15—C14—H14	120.6
C5—C6—H6	120.4	C13—C14—H14	120.6
C6—C7—C2	122.6 (3)	C10—C15—C14	122.2 (3)
C6—C7—H7	118.7	C10—C15—H15	118.9
C2—C7—H7	118.7	C14—C15—H15	118.9
C9—C8—H8A	109.5		

### Hydrogen-bond geometry (Å, °)

**Table:**

*D*—H···*A*	*D*—H	H···*A*	*D*···*A*	*D*—H···*A*
N1—H1···O2	0.86	1.95	2.637 (3)	136
O2—H2···O1^i^	0.82	1.86	2.677 (3)	178

Symmetry codes: (i) *x*, −*y*+1, *z*−1/2.
